# Editorial: Cardio-oncology: mechanisms and therapeutics

**DOI:** 10.3389/fcvm.2023.1198617

**Published:** 2023-05-03

**Authors:** Yan Ma, Feng Cao, Dong Han

**Affiliations:** National Clinical Research Center for Geriatric Diseases, The Second Medical Center, Chinese PLA General Hospital, Beijing, China

**Keywords:** cardio-oncolody, editoral, mechanisms, cardiotoxcity, prognosis

**Editorial on the Research Topic** Cardio-oncology: mechanisms and therapeutics

Cancer and cardiovascular disease share similar risk factors and are both prevalent among aging populations. Individuals with a history of cancer are exposed to a 2–3 times higher chance of getting acute coronary syndrome (ACS), which can persist for up to 10 years after a cancer diagnosis (1). Cancer patients with cardiovascular comorbidity have worse survival rates than cancer patients alone (2, 3). Reciprocally, myocardial infarction also hastens the spread of cancer and worsens the prognosis of cancer patients (4, 5). In this regard, understanding the interaction between cancer and cardiovascular disease may help avoid tackling diseases in a siloed approach and improve the outcome of these patients with comorbidity.

In addition, novel cancer therapies have tremendously improved the survival of cancer patients but also increased treatment-related side effects (6, 7). Cardiovascular toxicities are the most common adverse effects, threatening survival and impairing life quality of the cancer survivors (8). Cancer survivors' early morbidity and death are largely affected by these side effects (9). Understanding the mechanisms underlying anticancer treatment-induced cardiotoxicity can help develop novel therapeutics to avoid or lessen it.

The purpose of this research topic is to bring together a collection of works that provide novel insights into interactions between cancer and cardiovascular disease as well as mechanisms and therapeutics of anticancer treatment-induced cardiotoxicity. All contributions to this research topic concentrate on one or more of the above-mentioned study topics and several studies referenced below are representative.

## N^6^-Methyladenosine in cyclophosphamide-induced cardiotoxicity

The RNA epitranscriptomics represented by N^6^-Methyladenosine (m^6^A) are increasingly recognized to play important roles in physiology and disease (10). Cyclophosphamide is frequently prescribed to treat various types of cancers and autoimmune conditions. Accumulated doses of this drug may result in fatal hemorrhagic myocarditis (11). Zhu et al. demonstrated that the pathogenesis of cyclophosphamide-induced cardiotoxicity involves the downregulation of Junctophilin 2 (JPH2) levels. The proper structure and function of junctophilin-2 (JPH2) are recognized to be indispensable for proper excitation-contraction coupling in cardiomyocytes (12). The increased m^6^A level of JPH2 mRNA induced by N^6^-Methyladenosine writer METTL3 decreased its expression levels, and consequently dysregulated calcium signaling in cardiomyocytes. These results identified a novel epitranscriptomic mechanism regulating JPH2 expression and offers novel approaches to the management of cyclophosphamide-induced cardiotoxicity.

## miR-194-5p contributes to doxorubicin-induced cardiotoxicity

Doxorubicin is a popular anticancer agent but is well-known for its cardiotoxicity in many patients. The mechanisms underlying doxorubicin (DOX)-induced cardiotoxicity remain not fully understood. miRNAs are widely involved in the progression of various cardiovascular diseases (13). Fa et al. revealed the important role of miR-194-5p in the pathogenesis of DOX-induced cardiotoxicity. MiR-194-5p silencing reduced doxorubicin (DOX)-induced cardiotoxicity *in vitro* and *in vivo* by upregulating PAK2 and XBP1s. Overexpression of PAK2 or XBP1s reduced miR-194-5p- exacerbated cardiomyocyte apoptosis. This work was the first to identify a novel pathogenic miR-194-5p/PAK2/XBP1s axis in DOX-induced cardiotoxicity, hence proposing a potential target for the prevention and treatment of DOX-induced cardiotoxicity.

## NT-proBNP can predict cardiovascular symptoms caused by Pd-1 inhibitor therapy

In recent years, immunotherapy has achieved great success in cancer treatment. Unfortunately, cardiotoxicity appears to have emerged as an unneglectable issue recently (14). The work by Peng et al. suggested that NT-proBNP could predict cardiovascular symptoms in individuals with myocardial damage following PD-1 inhibitor therapy, while highly sensitive troponin T (hsTnT) is the best cardiac biomarker for mortality prediction in symptomatic patients. This study may help medics to perform risk stratification for patients at an earlier time and to implement effective interventions at the early stage of PD-1 inhibitor-related myocarditis.

## A large-scale observation in cancer patients suffering from infective endocarditis

Infective endocarditis (IE) occurs more frequently in cancer patients as compared with the general population (15). IE was predominantly community-acquired (74.8%) in cancer patients, according to Cosyns et al. The most common complications were acute renal failure (25.9%), embolic events (21.7%), and congestive heart failure (18.1%). This is a sizable observational cohort of IE patients with cancer. It sheds light on current IE cancer patient profiles, treatment, and outcomes. Considering the lack of randomized and large-scale observational data on IE cancer patients, this registry provides a unique viewpoint on IE management in cancer patients.

## D-Dimer is a predictive factor for cancer therapeutics-related cardiac dysfunction

Improved early detection methods have allowed a larger number of cancer patients with cancer therapeutics-related cardiac dysfunction (CTRCD) to live longer (16). Oikawa et al. consecutively enrolled 169 patients who planned to receive cardiotoxic chemotherapy for 12 months of follow-up and found that the incidence of CTRCD was greater in the high D-dimer group than in the low D-dimer group (16.2 vs. 4.5%, *p* = 0.0146). High D-dimer levels at baseline were an independent predictor of the development of CTRCD, according to multivariable logistic regression analysis [odds ratio 3.93, 95% CI (1.00–15.82), *p* = 0.047]. It is suggested that D-dimer may be a potential predictor of CTRCD and has clinical practical value.

## Low LVEF after chemotherapy was associated with blood RNA viruses

It has been hypothesized that immunosuppression after chemotherapy increases opportunistic viral infections (17). Varkoly et al. performed high-throughput sequencing analysis of RNA obtained from blood samples of 28 patients with hematological malignancies who had undergone chemotherapy. The result suggested that patients with low LVEF had influenza orthomyxovirus, avian paramyxovirus, and retrovirus sequences present. This is the first study to use high-throughput, blinded, unbiased sequencing to test for RNA viruses in circulating blood and associate those findings with abnormalities in heart function in patients who have recently finished chemotherapy. This study raises attention to RNA virus infections in individuals with chemotherapy-related cardiomyopathy.

## Cardiovascular outcomes in patients with colorectal cancer

Colorectal cancer (CRC) patients are potentially at high cardiovascular risk (18). Hang et al. followed up 197, 699 colorectal cancer patients for 37 months and examined the risks of cardiovascular death (CVD) in patients with CRC. They revealed that CVD ranked first and accounted for 41.69% of the major cause of non-cancer deaths. In addition, the nomogram for CVD prediction in CRC patients was created. This nomogram performed quite well and might assist physicians in providing customized care in clinical settings.

## Perspectives

With the generous support from all editors, publishers, reviewers, and authors involved in this research topic, we have successfully finalized this wonderful collection focusing on mechanisms and therapeutic in Cardio-Oncology. Future studies on the mechanism and management of cardio-oncology are expected to continually improve the survival and life quality of cancer survivors. The enormous issues posed by tumor-cardiovascular comorbidity, however, deserve more attention given its rising incidence and the continuously aging population. There is substantial opportunity for the collaboration between oncologists and cardiologists to work together to improve the outcome of cancer patients with cardiovascular comorbidity.
